# High-*T*_g_, Low-Dielectric Epoxy Thermosets Derived from Methacrylate-Containing Polyimides

**DOI:** 10.3390/polym10010027

**Published:** 2017-12-25

**Authors:** Chien-Han Chen, Kuan-Wei Lee, Ching-Hsuan Lin, Ming-Jaan Ho, Mao-Feng Hsu, Shou-Jui Hsiang, Nan-Kun Huang, Tzong-Yuan Juang

**Affiliations:** 1 Department of Chemical Engineering, National Chung Hsing University, Taichung 402, Taiwan; jain6792002@gmail.com (C.-H.C.); tgdyt547165@gmail.com (K.-W.L.); 2 Zhen Ding Technology Holding Limited, Taoyuan 33754, Taiwan; jay.mj.ho@zdtco.com (M.-J.H.); david.sj.hsiang@mail.zdtco.com (S.-J.H.); ryan.nk.huang@mail.zdtco.com (N.-K.H.); 3 Department of Cosmeceutics, China Medical University, Taichung 404, Taiwan; brian.mf.hsu@zdtco.com (M.-F.H.); tyjuang@mail.cmu.edu.tw (T.-Y.J.)

**Keywords:** epoxy, methacrylate, polyimide, low-dielectric, thermoset

## Abstract

Three methacrylate-containing polyimides (Px–MMA; x = 1–3) were prepared from the esterification of hydroxyl-containing polyimides (Px–OH; x = 1–3) with methacrylic anhydride. Px–MMA exhibits active ester linkages (Ph–O–C(=O)–) that can react with epoxy in the presence of 4-dimethylaminopyridine (DMAP), so Px–MMA acted as a curing agent for a dicyclopentadiene-phenol epoxy (HP7200) to prepare epoxy thermosets (Px–MMA/HP7200; x = 1–3) thermosets. For property comparisons, P1–OH/HP7200 thermosets were also prepared. The reaction between active ester and epoxy results in an ester linkage, which is less polar than secondary alcohol resulting from the reaction between phenolic OH and epoxy, so P1–MMA/HP7200 are more hydrophobic and exhibit better dielectric properties than P1–OH/HP7200. The double bond of methacrylate can cure at higher temperatures, leading to epoxy thermosets with a high-*T*_g_ and moderate-to-low dielectric properties.

## 1. Introduction

Epoxy thermosets exhibit good chemical resistance, dimensional stability, insulation, and adhesion properties, so they are widely used in such electronic fields as encapsulation and printed circuit boards [1,2,3]. Epoxy resins are commonly cured by active hydrogen-containing compounds such as phenol novolac, bisphenol A novolac, dicyandiamide, and diamine [4,5,6,7,8,9,10,11,12,13]. However, the reaction product from active hydrogen and epoxy lead to a secondary alcohol. This alcohol is a highly polar group, resulting in epoxy thermosets with a high dielectric constant and dissipation factor. To avoid the formation of secondary alcohol, anhydride-type [11,12,14,15,16] or active ester-type epoxy curing agents [17,18,19] are applied. Researchers at Isola used a styrene maleic anhydride (SMA) copolymer as a curing agent to achieve epoxy thermosets with better dielectric properties [20]. A commercial product EPICLON HPC-8000-65T, which is a dicyclopentadiene phenol novolac-based active ester, has been commercialized by DIC [21]. Other products such as DC808 and YLH1026, which are phenol novolac-based active esters, have been commercialized by Japan Epoxy Resins [22].

Nakamura et al. prepared epoxy thermosets using a triphenol and its active esters [18]. They found that the dielectric constant of an active ester-cured epoxy thermoset is lower than the triphenol-cured thermoset. However, according to Nakamura’s result, the *T*_g_ of active esters-cured epoxy thermoset is lower than triphenol-cured thermoset because of the reduced intermolecular interaction. Takeuchi et al. also found that an epoxy thermoset cured by a benzoyl chloride-modified dicyclopentadiene-phenol active ester exhibited a much lower *T*_g_. However, they also found that the reduction in *T*_g_ in an epoxy thermoset cued by an isophthalic chloride-modified dicyclopentadiene-phenol active ester was not obvious [23]. That is, the reduction in *T*_g_ in an active ester-cured epoxy thermoset can be compensated through the increasing molecular weight of curing agent. However, from a theoretical view point, gel might occur in this multifunctional system.

Generally, epoxy thermosets are relatively brittle and display poor resistance to crack propagation. Some approaches have been taken to increase the toughness of epoxy thermoset, for example: (1) incorporating rubber such as carboxylic acid terminated butadiene acrylonitrile rubber (CTBN), epoxy-terminated butadiene acrylonitrile (ETBN), amine-terminated butadiene acrylonitrile (ATBN), and hydroxyl-terminated polybutadiene (HTPB) into the epoxy matrix [24]; (2) toughening the epoxy thermoset through the self-assembling of amphiphilic block copolymers into vesicles and micelles has been reported [25,26,27]; (3) blending epoxy with engineering thermoplastics, such as poly(phenylene oxide)s, poly(ether sulfones)s [28], poly(ether imide)s [29], polyamides [30], and polycarbonates [31,32] has been reported; and (4) incorporating functionalized thermoplastics, such as amine-terminated poly(aryl ether ketone) [33], amine functionalized poly(aryl sulfone) [34], and phenol-functionalized polyether [35,36], which behave as curing agents for epoxy resins and promote interfacial bonding between the thermoplastic and thermoset through the formation of covalent linkage has also been discussed.

Polyimides are widely used in fields that require high thermal stability, excellent mechanical properties and chemical resistance such as flexible printed circuit boards. Research on polyimides/epoxy composites has been reported in literature. For example, Kakimoto et al. [37] blended diglycidyl ether of bisphenol A (DGEBA) with a poly(amic acid) derived from oxydianiline and pyromellitic dianhydride (PMDA), followed by thermal imidization. They found that the adhesive qualities of the epoxy were dramatically improved with the incorporation of a poly(amic acid) as the curing agent. The reaction between the epoxy and the hydroxyl functionalities present on the poly(hydroxyl imide) results in the formation of a network structure. TGA data showed that polyimide/epoxy thermosets are thermally more stable than the diamine/epoxy thermosets. To avoid shrinkage during curing encountered with using the poly(amic acid)/epoxy system, Takeichi et al. took another approach to prepare epoxy/polyimide composites by using soluble reactive poly(hydroxyl imide) derived from 2,2-bis(3,4-dicarboxyphenyl)hexafluoropropane dianhydride (6FDA) and 3,3′-diamino-4,4′-dihydroxybiphenyl as a hardener [38]. The glass transition temperature, tensile modulus, solvent resistance, and thermal stability were enhanced with the incorporation of polyimide. Other research on polyimide and epoxy has also been reported. Park et al. prepared a polyimide-graft-bisphenol A diglyceryl acrylate as a dispersant of carbon nanotubes from the reaction of glycidyl terminated bisphenol A acrylate hydroxyl and a hydroxyl group of poly(hydroxyl imide), which is derived from 4,4′-oxydiphthalic anhydride (ODPA) and 3,3′-dihydroxy-4,4′-diaminobiphenyl [39]. Horie et al. synthesized an epoxy-containing polyimide from the epoxidation of a poly(hydroxyl imide) derived from 6FDA and 2,2-bis(3-amino-4-hydroxyphenyl)hexafluoropropane (AHHFP) [40]. The epoxy-containing polyimide, combined with an acid generator diphenyliodonium hexafluoroarsenate, was found to work as a negative-tone photosensitive polyimide with high sensitivity and contrast.

To the best of our knowledge, no methacrylate-containing polyimide/epoxy thermosets have been reported. In this work, three methacrylate-containing polyimides (Px–MMA, x = 1–3) were prepared from three hydroxyl-containing polyimides (Px–OH, x = 1–3). Px–MMA exhibits active ester linkages (Ph–O–C(=O)–) that can react with the epoxy in the presence of 4-dimethylaminopyridine (DMAP), so Px–MMA acted as a hardener for a dicyclopentadiene-phenol epoxy to prepare epoxy thermosets (Px–MMA/HP7200, x = 1–3). For property comparisons, P1–OH/HP7200 was also prepared. Experimental data show that P1–MMA/HP7200 thermosets are more hydrophobic and exhibit better dielectric properties than P1–OH/HP7200. Detailed synthesis and analysis, curing mechanism, and the structure-property relationship of the resulting epoxy thermosets are provided in this work.

## 2. Experimental

### 2.1. Materials

9,10-Dihydro-oxa-10-phosphaphenanthrene-10-oxide (DOPO, TCI, Shanghai, China), 4-aminoacetophenone (Acros, NJ, USA), 2-aminophenol (Acros, New Jersey, USA), *p*-toluene sulfonic acid monohydrate (p-TSA, Showa, Tokyo, Japan), 2,2-bis (3-amino-4-hydroxyphenyl) propane (TCI), 2,2-bis (3-amino-4-hydroxyphenyl) hexafluoropropane (TCI), 4-dimethylaminopyrdine (DMAP, Alfa, Heysham, UK), sodium acetate (Showa), methacrylic anhydride (Alfa), t-butyl cumyl peroxide (TBCP, Sigma-Aldrich, St. Louis, MO, USA) and dicyclopentadiene-phenol epoxy (HP-7200, EEW = 250 g/eq, DIC, Tokyo, Japan) were used as received. 4,4′-oxydiphthalic anhydride (ODPA, Chriskev, Lenexa, KS, USA) and 2,2-bis (3,4-dicarboxyphenyl) hexafluoropropane dianhydride (6FDA, Chriskev, Lenexa, KS, USA) were recrystallized from acetic anhydride. The solvents used are commercial products (HPLC grade) and were used without further purification.

### 2.2. Characterization

NMR spectra were obtained by a Varian Inova 600 NMR (Agilent, Palo Alto, CA, USA) in CDCl3 or in Dimethyl sulfoxide-*d_6_* (DMSO-*d*_6_). Differential scanning calorimetry (DSC) was performed with a Perkin-Elmer DSC 8000 (Perkin-Elmer, Shelton, CT, USA) in a nitrogen atmosphere at a heating rate of 20 °C/min. Dynamic mechanical analysis (DMA) was performed with a Perkin-Elmer Pyris Diamond DMA (Perkin-Elmer, Shelton, CT, USA). The storage modulus *E*′ and tan δ were determined as the sample was subjected to a temperature scan mode at a programmed heating rate of 5 °C/min at a frequency of 1 Hz. The test was performed by tension mode with an amplitude of 25 μm. Contact angle was performanced with FTA1000B (First Ten Angstroms, Portsmouth, VA, USA) through the Sessile drop method and used water as a testing solvent. Dielectric properties were performed with Agilent E4991A (Agilent, Santa Clara, CA, USA) at 1 GHz at 25 °C. The sample size is 2 × 2 cm^2^ with thickness at around 300 µm. IR Spectra were obtained in the standard wavenumber range of 400–4000 cm^−1^ by a Perkin-Elmer RX1 infrared spectrophotometer (Perkin-Elmer, Shelton, CT, USA). Thermal mechanical analysis (TMA) was performed by a SII TMA/SS6100 (Seiko, Torrance, CA, USA) at a heating rate of 5 °C/min. The coefficient of thermal expansion (CTE) was determined in the range of 50 to 150 °C. Thermal gravimetric analysis (TGA) was performed by a Perkin-Elmer Pyris 1 TGA (Perkin-Elmer, Shelton, CT, USA) in a nitrogen atmosphere or in an air atmosphere from 50 to 800 °C at a heating rate of 20 °C/min. Gel Permeation Chromatography (GPC) was performed by Hitachi L2400 in *N*-methyl-2-pyrrolidone (NMP) (Hitachi, Tarrytown, NY, USA), using polystyrene as standard.

### 2.3. Synthesis of Hydroxyl Diamine

The synthesis of hydroxyl diamine was performed according to literature [41]. DOPO 4.80 g (22.21 mmol), 4-aminoacetophenone 3.0 g (22.21 mmol), 2-aminophenol 4.84 g (44.42 mmol), *p*-TSA 0.192 g (4 wt.. % based on DOPO) and DMSO 10 mL were introduced into a 100-mL round-bottom glass flask equipped with a nitrogen inlet and a magnetic stirrer. The reaction mixture was stirred at 100 °C for 12 h. After that, the precipitate was washed several times with methanol, recrystallized from methanol, and then dried in a vacuum oven at 100 °C for 8 h. Light white crystals (68% yield) with a melting point of 281 °C (DSC) were obtained. ^1^H-NMR (DMSO-*d*_6_), 1.47 ppm (CH_3_), 4.45 ppm (NH_2_), 5.02 ppm (NH_2_), 6.38–8.08 ppm (Ar–H), 8.95 (OH).

### 2.4. Preparation of Poly(hydroxyl imide) P1–OH

Diamine (**1**) 1.0 g (2.26 mmole), 6FDA 1.004 g (2.26 mmole), NMP 8.0 g, and xylene 4.0 g were added to a 100-mL three-neck round-bottom flask equipped with a magnetic stirrer, a dean-stark apparatus, and a nitrogen inlet. The reaction was carried out at reflux temperature for 24 h. Then, the viscous polyimides solution was poured into methanol. The precipitate was filtered and dried at 80 °C overnight. Yield > 96%. ^1^H-NMR (DMSO-*d*_6_): 10.0 ppm (Ar–OH), 6.8–8.2 ppm (Ar–H), 1.6 ppm (CH_3_). ^31^P-NMR (DMSO-*d*_6_): 37.5 ppm. IR: 3280 cm^−1^ (OH), 1780 and 1720 cm^−1^ (imide), 1370 cm^−1^ (C–N–C), 1238 cm^−1^ (P=O).

### 2.5. Preparation of Poly(hydroxyl imide) P2–OH

2,2-Bis(3-amino-4-hydroxyphenyl)propane (**2**) 1.0 g (3.87 mmole), 6FDA 1.720 g (3.87 mmole), NMP 10.8 g, and xylene 5.4 g were added to a 100-mL three-neck round-bottom flask equipped with a magnetic stirrer, a dean-stark apparatus, and a nitrogen inlet. The reaction was carried out at reflux temperature for 24 h. Then, the viscous polyimides solution was poured into methanol. The precipitate was filtered and dried at 80 °C overnight. Yield > 97%. ^1^H-NMR (DMSO-*d_6_*): 10.0 ppm (Ar–OH), 6.8–8.2 ppm (Ar–H), 1.6 ppm (CH_3_). IR: 3280 cm^−1^ (OH), 1780 and 1720 cm^−1^ (imide), 1370 cm^−1^ (C–N–C), 1100 cm^−1^ (C–F).

### 2.6. Preparation of Poly(hydroxyl imides) P3–OH

2,2-Bis(3-amino-4-hydroxyphenyl) hexafluoropropane (**3**) 1.0 g (2.73 mmole ), 6FDA 1.200 g (2.73 mmole), NMP 8.8 g, and xylene 4.4 g were added to a 100-mL three-neck round-bottom flask equipped with a magnetic stirrer, a dean-stark apparatus, and a nitrogen inlet. The reaction was carried out at reflux temperature for 24 h. Then, the viscous polyimides solution was poured into methanol. The precipitate was filtered and dried at 80 °C overnight. Yield > 97%. ^1^H-NMR (DMSO-*d_6_*): 10.4 ppm (Ar–OH), 7.0–8.2 ppm (Ar–H). IR: 3280 cm^−1^ (OH), 1780 and 1720 cm^−1^ (imide), 1370 cm^−1^ (C–N–C), 1100 cm^−1^ (C–F).

### 2.7. Preparation of Poly(methacrylate imides) P1–MMA

P1–OH 1.00 g (1.18 mmol repeat unit), methacrylic anhydride 0.29 g (1.88 mmol), DMAP 0.0044 g and *N,N*-dimethylacetamide (DMAc) 10 mL were added to a 100-mL three-neck round-bottom flask equipped with a magnetic stirrer, and a nitrogen inlet. The reaction was carried out at room temperature for 24 h. Then, the solution was poured into methanol. The precipitate was filtered and dried at 80 °C overnight. Grey powder (94% yield) was obtained. ^1^H-NMR (DMSO-*d_6_*): 7.0–8.4 ppm (Ar–H); 5.8 and 6.2 ppm (–C=CH_2_); 1.9 and 1.6 ppm (CH_3_). ^31^P–NMR (DMSO–*d_6_*): 36.5 ppm. IR: 1780 and 1720 cm^−1^ (imide), 1238 cm^−1^ (P=O), 1370 cm^−1^ (C–N–C), 1105 cm^−1^ (Ar–O–C), 950 cm^−1^ (–C=C–H). GPC: number-average molecular weight 34,000 g/mol, weight-average molecular weight 71,000 g/mol.

### 2.8. Preparation of Poly(methacrylate imide) P2–MMA

P1–OH 1.00 g (1.50 mmol repeat unit), methacrylic anhydride 0.98 g (6.38 mmol), DMAP 0.015 g and DMAc 10 mL were added to a 100-mL three-neck round-bottom flask equipped with a magnetic stirrer, and a nitrogen inlet. The reaction was carried out at room temperature for 24 h. Then, the solution was poured into methanol. The precipitate was filtered and dried at 80 °C overnight. Grey powder (95% yield) was obtained. ^1^H-NMR (DMSO-*d_6_*): 7.2–8.2 ppm (Ar–H); 5.8 and 6.2 ppm (–C=CH_2_); 1.9 and 1.6 ppm (CH_3_). IR: 1780 and 1720 cm^−1^ (imide), 1370 cm^−1^ (C–N–C), 1105 cm^−1^ (Ar–O–C), 950 cm^−1^ (–C=C–H). GPC: number average-molecular weight 36,000 g/mol, weight-average molecular weight 75,000 g/mol.

### 2.9. Preparation of Poly(hydroxyl imides) P3–MMA

P3–OH 1.00 g (1.13 mmol repeat unit), methacrylic anhydride 0.85 g (5.53 mmol), DMAP 0.013 g and DMAc 10 mL were added to a 100-mL three-neck round-bottom flask equipped with a magnetic stirrer, and a nitrogen inlet. The reaction was carried out at room temperature for 24 h. Then, the solution was poured into methanol. The precipitate was filtered and dried at 80 °C overnight. Grey powder (94% yield) was obtained. ^1^H-NMR (DMSO-*d_6_*): 7.4–8.2 ppm (Ar–H); 5.8 and 6.2 ppm (–C=CH_2_); 1.9 ppm (CH_3_). IR: 1780 and 1720 cm^−1^ (imide), 1370 cm^−1^ (C–N–C), 1105 cm^−1^ (Ar–O–C), 950 cm^−1^ (C=C–H). GPC: number-average molecular weight 29,000 g/mol, weight-average molecular weight 61,000 g/mol.

### 2.10. Preparation of Px–MMA/HP7200 Thermosets

The same equivalence of Px–MMA (the equivalent weight for P1–MMA is the molecular weight of its repeating unit, while for P2–MMA and P3–MMA is half the molecular weight of its repeating unit) and HP7200 (epoxy equivalent weight 250 g/eq), along with DMAP 0.5 wt. % (based on HP-7200) and TBCP (1 wt. % based on Px–MMA) were dissolved in NMP to make a 30 wt. % solution. The solution was cast by an automatic film applicator. The film was dried at 100 °C overnight, and 180, 200, 220, 260 and 300 °C for 1 h each. The sample IDs are P1–MMA/HP7200, P2–MMA/HP7200, and P3–MMA/HP7200, respectively.

### 2.11. Preparation of P1–OH/HP7200 Thermosets

For property comparisons with the P1–MMA/HP7200 thermoset, a P1–OH/HP7200 thermoset was also prepared. The same equivalence of P1–OH (the equivalent weight for P1–OH is the molecular weight of its repeating unit) and HP7200 (epoxy equivalent weight 250 g/eq), along with DMAP 0.5 wt. % (based on HP-7200) were dissolved in NMP to make a 30 wt. % solution. The solution was cast by an automatic film applicator. The film was dried at 100 °C overnight, and 180, 200, 220, 260 and 300 °C for 1 h each. The sample ID is P1–OH/HP7200.

## 3. Results and Discussion

### 3.1. Synthesis of Px–OH and Px–MMA

**Scheme 1:**
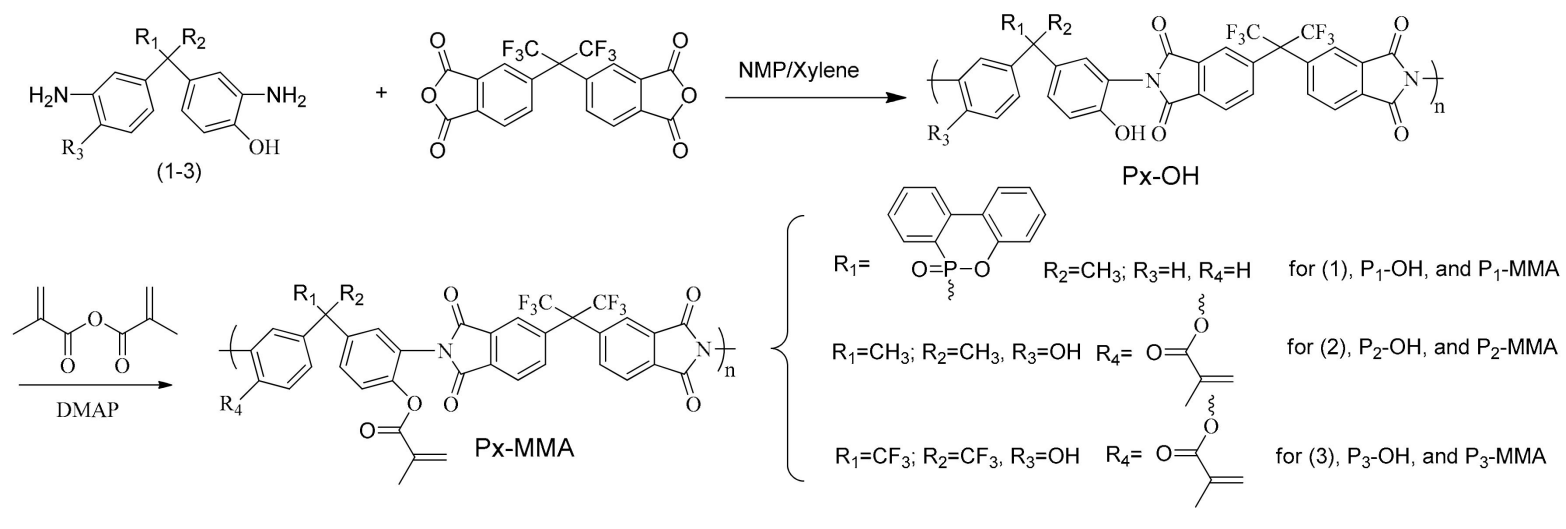
Synthesis of Px–OH and Px–MMA.

Hydroxyl-containing polyimides (Px–OH, x = 1–3) were prepared from the reaction of hydroxyldiamines (1–3) with dianhydride 6FDA in NMP/xylene by solution polymerization (Scheme 1). Methacrylate-containing polyimides (Px–MMA, x = 1–3) were prepared from the reaction of methacrylic anhydride with the phenolic group of Px–OH in the presence of catalyst. Table 1 shows the effect of the reaction conditions on P1–MMA synthesis. Using DMAP as a catalyst at 100 °C (Runs 1–2), and 80 °C (Run 5), and sodium acetate at 100 °C (Run 3), a phenolic OH signal was observed in the ^1^H-NMR spectrum. The phenolic might come from the unreacted phenolic or from the cleavage of ester linkage. No signal phenolic OH was found when sodium acetate was used as the catalyst at 80 °C (Run 4). However, signals from unknown side reactions were observed. When the reaction was catalyzed by DMAP at 50 °C (Runs 6–7), no phenolic OH was found, but small side reaction peaks were observed. When the temperature was reduced to 25 °C (Run 8), no signal of phenolic OH was found, and characteristic peaks of methacrylate at 6.2, 5.8, and 1.9 ppm were found. These results indicate that the esterification could be carried out successfully at room temperature. 

**Figure 1:**
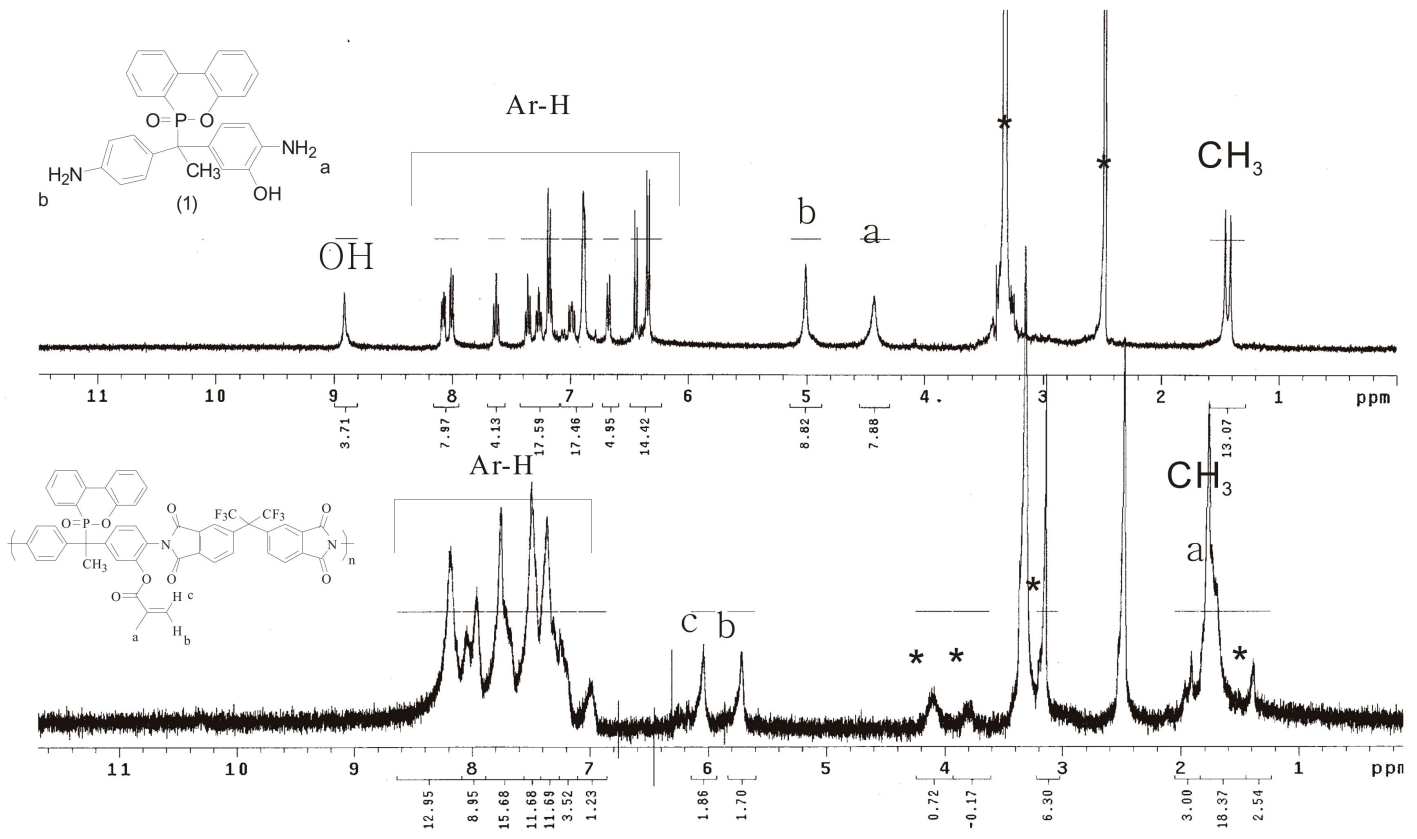
^1^H-NMR spectra of hydroxyl diamine (1) and P1–MMA in DMSO-*d_6_*. (*: Solvents).

**Figure 2:**
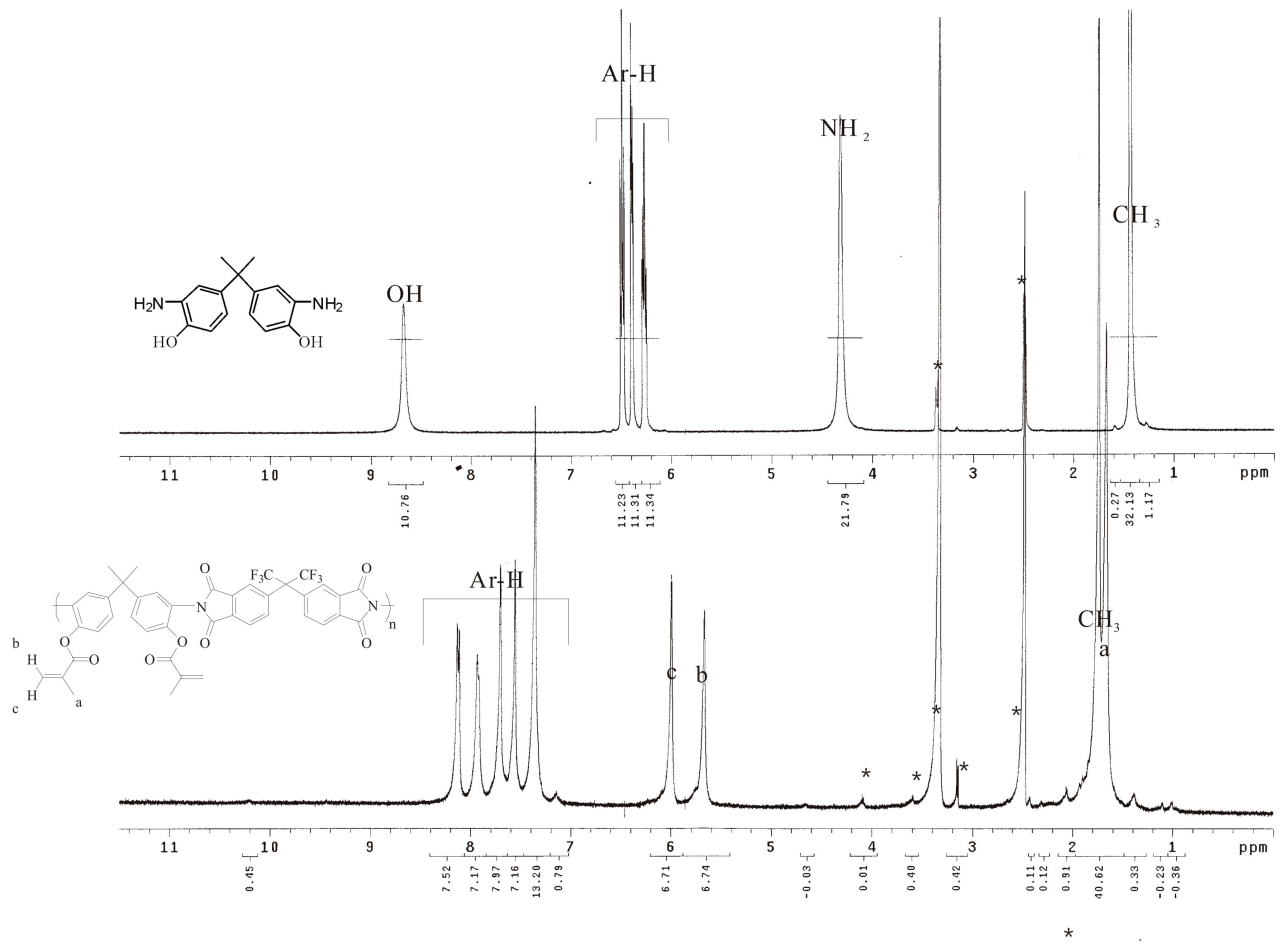
^1^H-NMR spectra of hydroxyl diamine (2) and P2–MMA in DMSO-*d_6_*. (*: Solvents).

**Figure 3:**
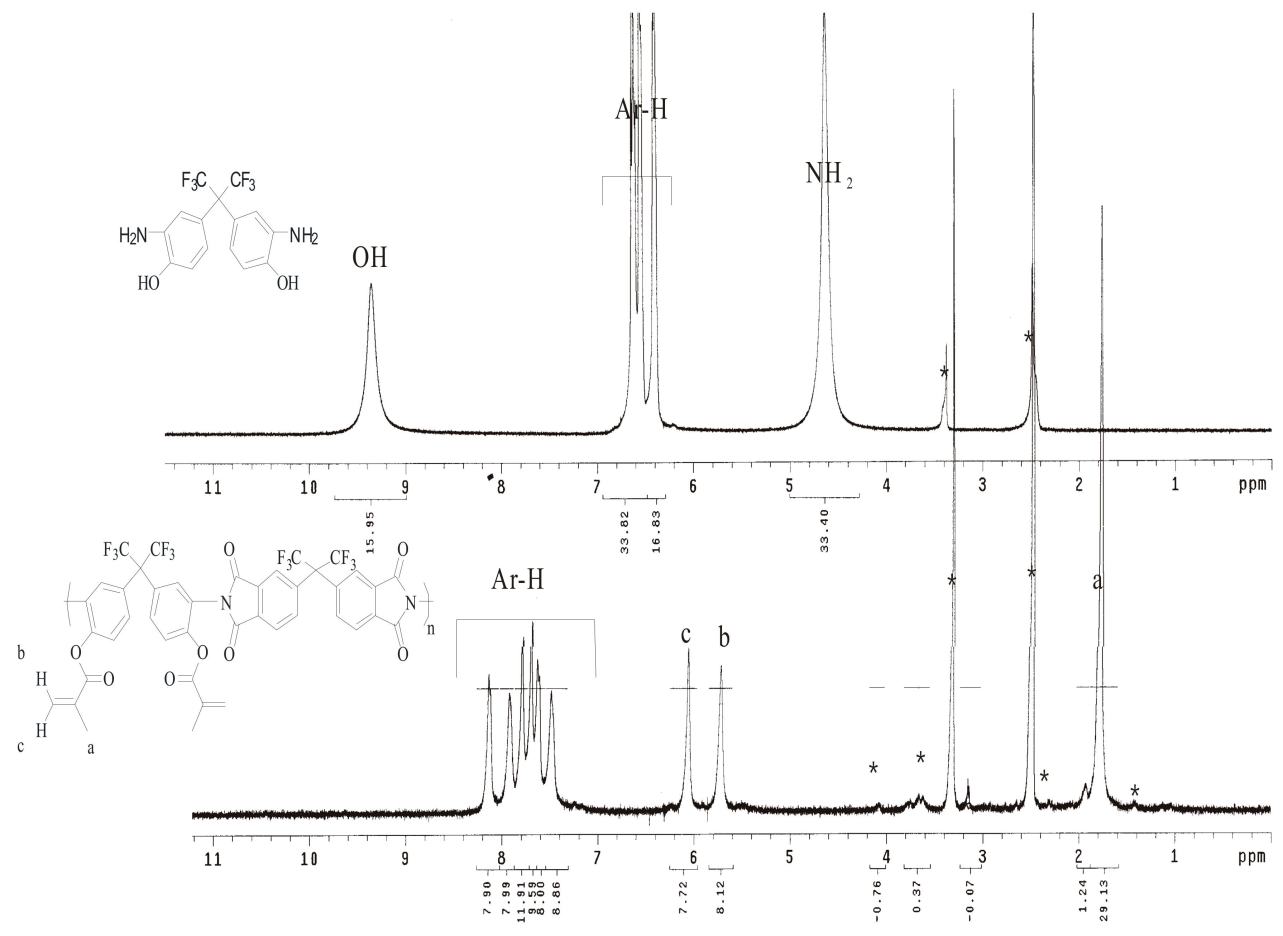
^1^H-NMR spectra of hydroxyl diamine (3) and P3–MMA in DMSO-*d_6_*. (*: Solvents).

Figure 1 shows the ^1^H-NMR spectra of hydroxyl diamine (1) and P1–MMA. The amine signals at 4.4 and 5.9 ppm and the phenolic signal at 8.9 ppm of hydroxyl diamine (1) disappear, and characteristic peaks for methacrylate of P1–MMA at 6.2, 5.8, and 1.9 ppm were found. Figure 2 shows the ^1^H-NMR spectra of hydroxyl diamine (2) and P2–MMA. Figure 3 shows the ^1^H-NMR spectra of hydroxyl diamine (3) and P3–MMA. The amine and hydroxyl signals disappeared, and new signals corresponding to methacrylate appeared in both Figure 2 and Figure 3. NMR data show the Px–MMA has successfully prepared.

### 3.2. DSC Thermograms of Px–MMA

**Figure 4:**
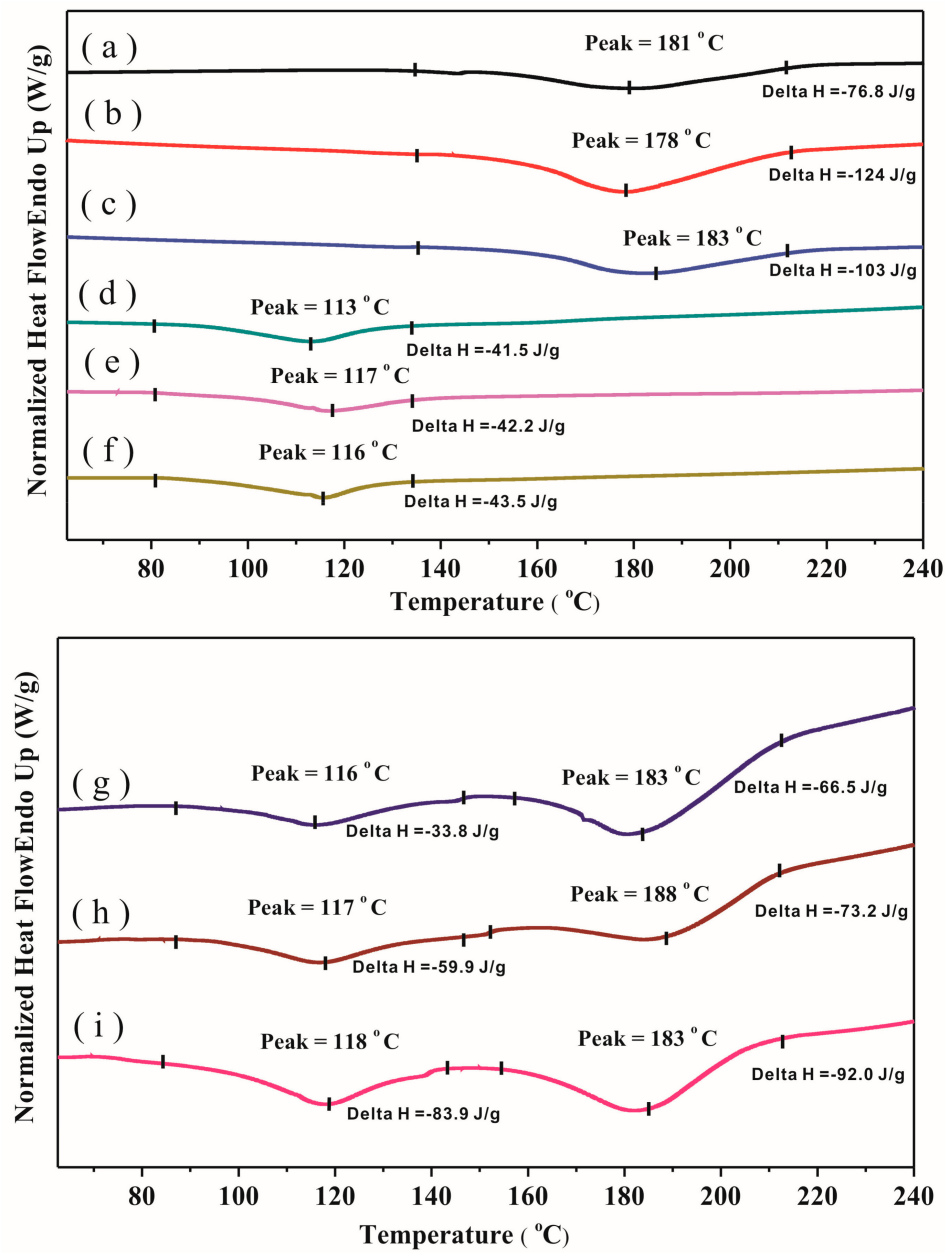
DSC thermograms of (**a**–**c**) Px–MMA (x = 1–3) with 1.0 wt.. % TBCP, (**d**–**f**) Px–MMA/HP7200 blend (x = 1–3) with 0.5 wt. % DMAP, and (**g**–**i**) Px–MMA (x = 1–3) with 1.0 wt. % TBCP and 0.5 wt.. % DMAP.

**Scheme 2:**
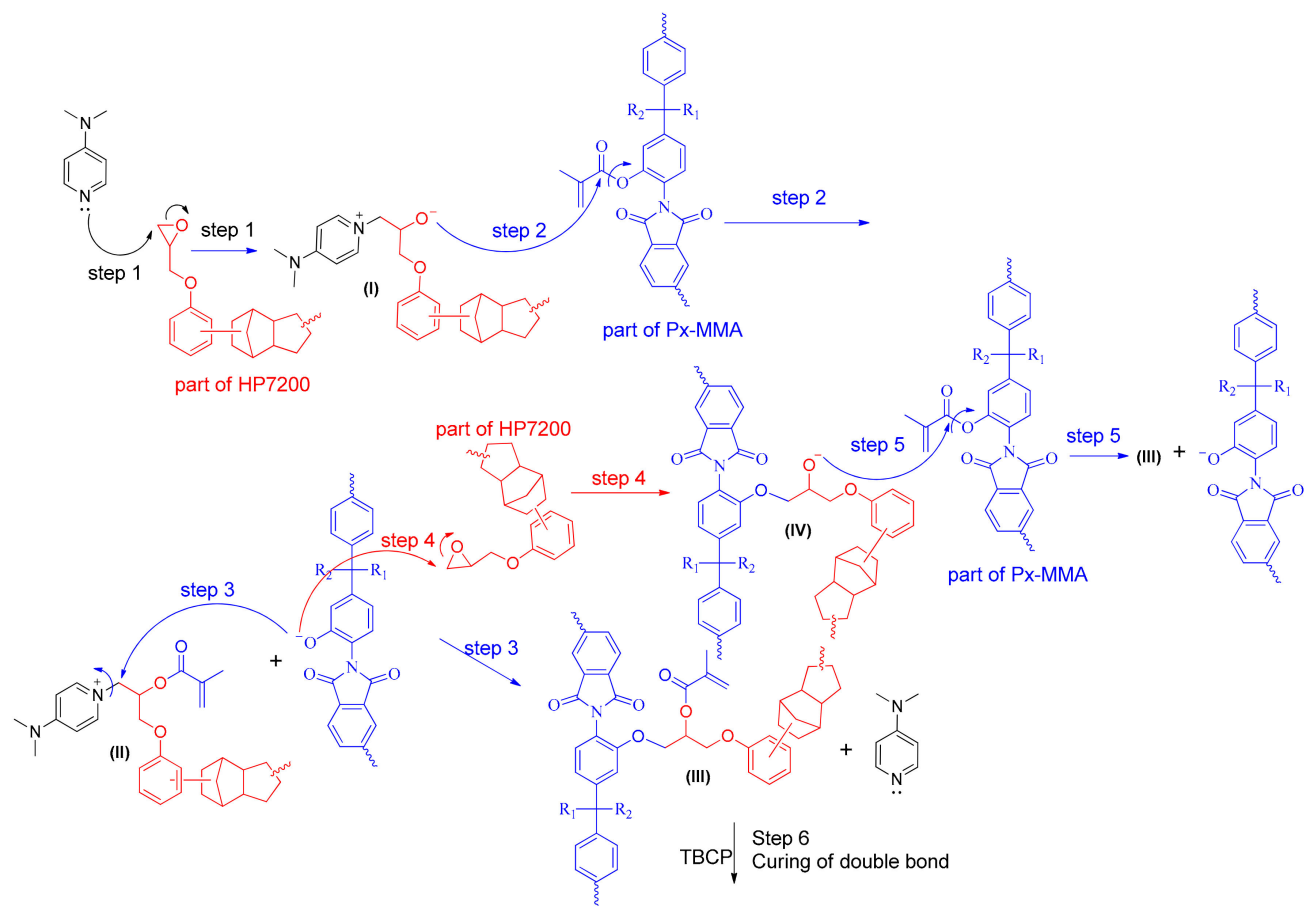
Reaction mechanism of HP7200 and Px–MMA in the presence of DMAP and TBCP.

Figure 4a–c shows the DSC heating scans of Px–MMA (x = 1–3) with 1.0 wt. % peroxide TBCP. An exothermic peak corresponding to the curing of double bond of methacrylate at around 180 °C was observed. Figure 4d–f show the DSC heating scans of Px–MMA/HP7200 blend (x = 1–3) with 0.5 wt. % DMAP. An exothermic peak at around 115 °C was observed. According to our previous study in the reaction of methacrylate and epoxy [42], we think that the exothermic peak is related to the DMAP-initiated ring-opening of epoxy, forming an alkoxy anion which then attack the ester group (Detailed mechanism will be discussed in Scheme 2). Figure 4g–i show the DSC heating scans of Px–MMA (x = 1–3) with 1.0 wt. % peroxide TBCP and 0.5 wt.. % DMAP. Two exothermic peaks at around 115 and 180 °C were observed in the DSC thermograms. Therefore, the curing sequence is thought to be the reaction of the DMAP/epoxy/active ester, followed by curing of the methacrylate moiety.

### 3.3. Proposed Mechanism

In our previous work [42], we reacted glycidyl phenyl ether and phenyl acetate in the presence of DMAP. The reaction product is exclusively 1,3-diphenoxy-2-acetoxypropane (no homopolymerizaiton product of glycidyl phenyl ether takes place). We also reacted glycidyl phenyl ether and phenyl methacrylate in the presence of DMAP. The reaction product is exclusive 1,3-diphenoxy-2-methacryalatepropane (no homopolymerizaiton product of glycidyl phenyl ether takes place, and no 1,4-conjugated nucleophilic addition to C=C–(C=O) or nucleophilic addition on the double bond of methacrylate occurs). based on the finding, we propose a reaction mechanism of HP7200 and Px–MMA in the presence of DMAP and TBCP. The first step is the attack of DMAP on the epoxy group of HP7200, forming intermediate (**I**) with an alkoxy anion. The second step is the nucleophilic substitution of the alkoxy anion of (**I**) on the ester group of Px–MMA exclusively, forming intermediate (**II**) and polyimide with a phenoxy anion (Note that no 1,4-conjugated nucleophilic addition on C=C–(C=O) or nucleophilic addition on the double bond of methacrylate occurs, as supported by the result of model reaction 3 [42] and the exothermic peak at around 180 °C due to the curing double bond in Figure 4g–i The third step is the nucleophilic substitution of the phenoxy anion of polyimide on the carbon of the C–N bond of (**II**), forming intermediate (**III**) and releasing DMAP that can repeat step 1. Another way to obtain (**III**) might occur through steps 4 and 5. The fourth step is the nucleophilic addition of the phenoxy anion of polyimide on the epoxy of HP7200, forming intermediate (**IV**) with an alkoxy anion. The fifth step is the nucleophilic substitution of alkoxy anion of (**IV**) on ester group of Px–MMA, forming (**III**) and polyimide with a phenoxy anion that can repeat step 4. The sixth step is TBCP-initiated thermal curing of methacrylate. 

**Figure 5:**
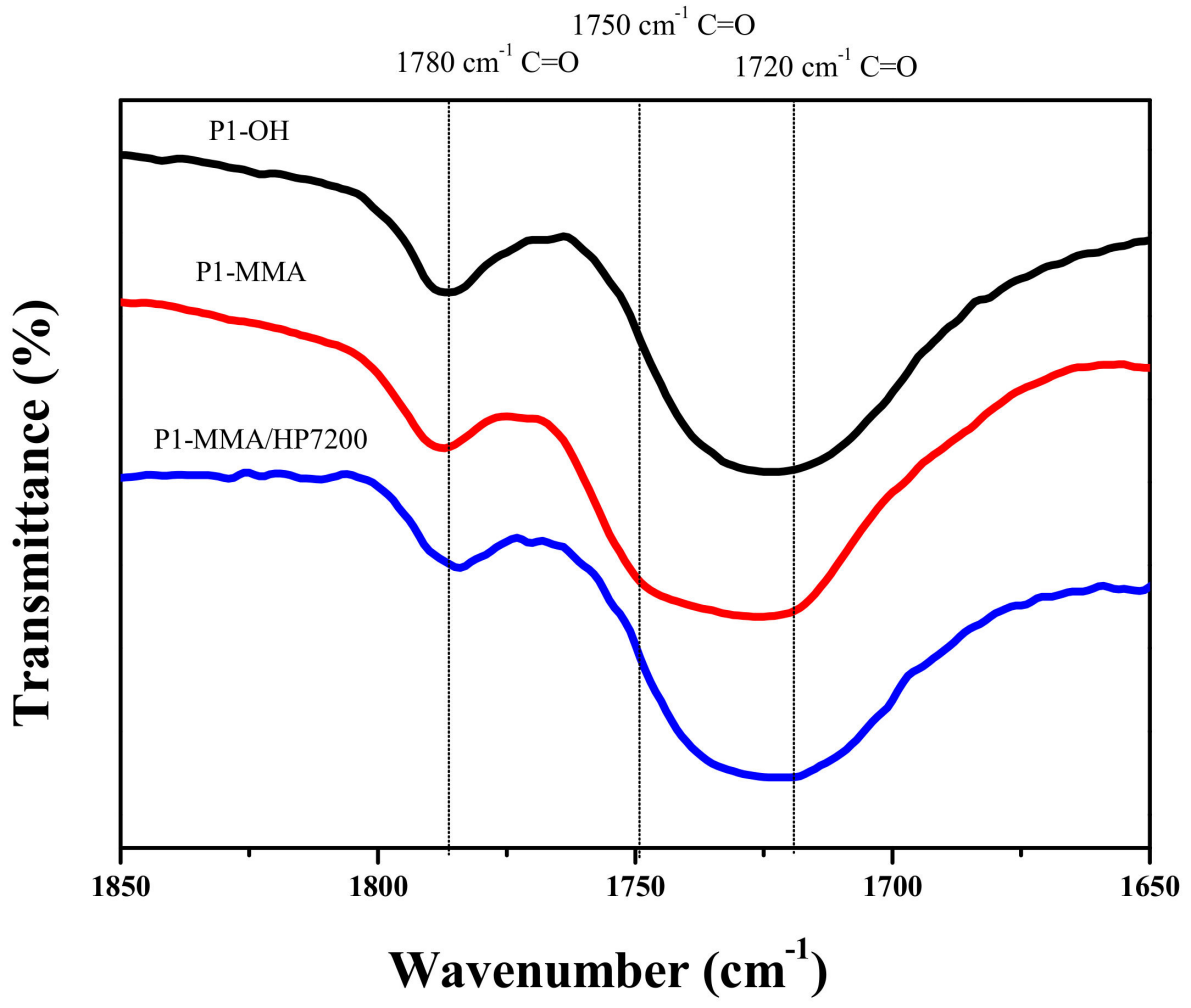
Enlarged IR spectra of P1–OH, P1–MMA, and P1–MMA/HP7200.

Figure 5 shows enlarged IR spectra of P1–OH, P1–MMA, and P1–MMA/HP7200. We can find that the imide absorptions are 1780 and 1720 cm^−1^ for P1–OH. Besides the imide absorptions at 1780 and 1720 cm^−1^, there is a shoulder peak at 1750 cm^−1^ for active ester, ph–O–(C=O), for P1–MMA. However, the shoulder peak upshift to 1730 cm^−1^ in P1–MMA/HP7200. The shift can be explained by the IR absorption principle that the ester absorption of ph–O–(C=O) has higher absorption than that of R–O–(C=O) [43]. The IR spectra support the proposed mechanism in Scheme 2.

### 3.4. Photo of Prepared Polymers

**Figure 6:**
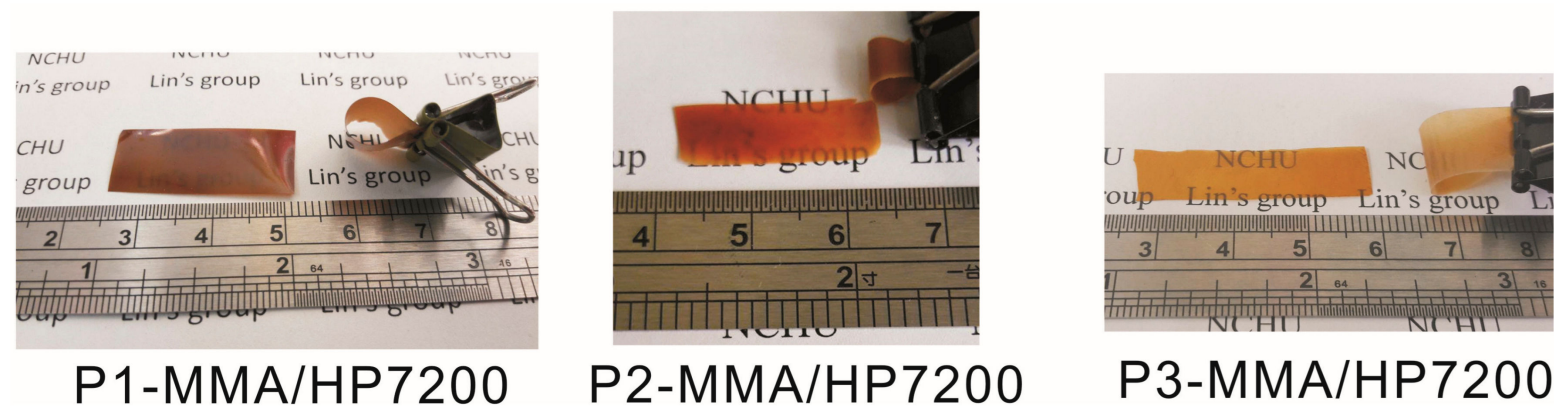
Photos of Px–MMA/HP7200.

Figure 6 shows photos of Px–MMA/HP7200. All of the films are homogeneous and tough, and can be bent without breaking. The flexibility of epoxy thermosets supports our previous result that epoxy thermosets cured by high molecular weight curing agent are flexible [35,36]. The homogeneous appearance of the film indicates that Px–MMA and HP7200 can react with each other, respectively. In addition, solubility test shows that all the Px–MMA/HP7200 films are insoluble, further supporting their network structures. Since all of the films are flexible, a dynamic mechanical analyzer was applied to measure their dynamic mechanical properties in a tension mode.

### 3.5. DMA and TMA Thermograms

**Figure 7:**
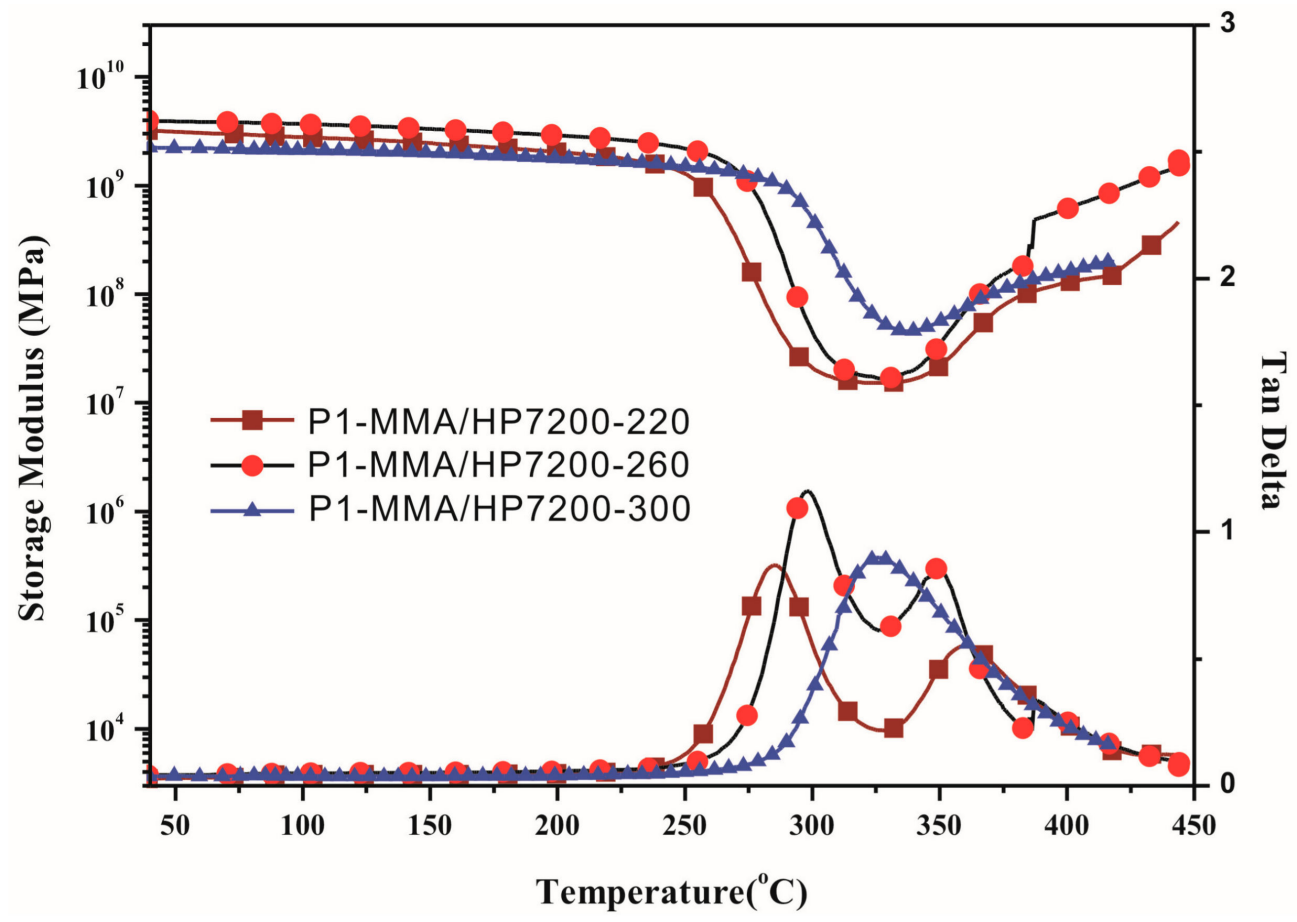
DMA thermogram of P1–MMA/HP7200 under three different curing temperature.

Figure 7 shows the DMA thermograms of P1–MMA/HP7200 under three final curing temperatures: 220 °C (1 h), 260 °C (1 h), and 300 °C (1 h), respectively. The number marked in Figure 7 is the final curing temperature. For 220 and 260 °C curing systems, two tan δ peaks were observed. The modulus decreased gradually when the temperature rises higher than 250 °C, reaches a minimum at around 320 °C, and increases gradually after 320 °C. This phenomenon indicates that the curing reaction at 220 or 260 °C is not complete. The molecules devitrificate and the curing process continues during the thermal scan. A single tan δ peak was observed until the final curing temperature was 300 °C (1 h), suggesting a higher conversion. A further higher curing temperature might lead to higher conversion. However, considering the potential thermal degradation at higher curing temperature, we set the final curing temperature to be 300 °C (1 h) for all systems. 

**Figure 8:**
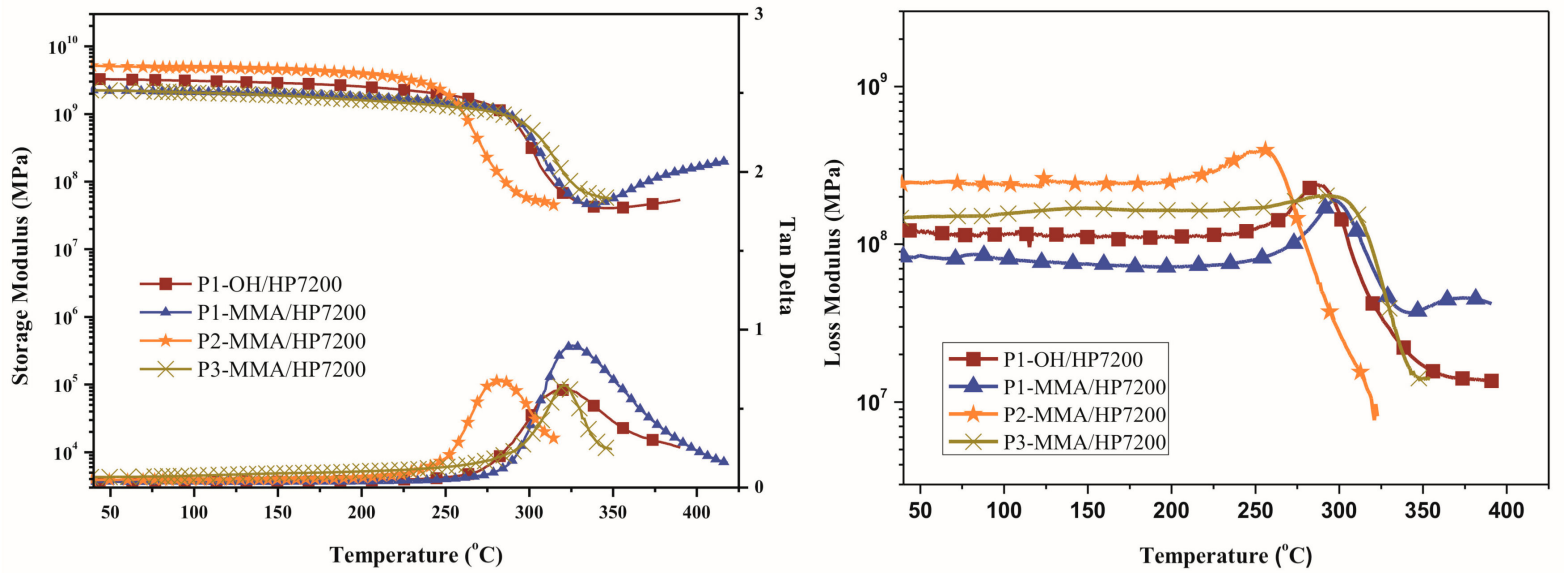
DMA thermograms of the prepared thermosets.

Figure 8 shows the DMA thermograms of the Px–MMA/HP7200 thermosets, and the results are listed in Table 2. The *T*_g_ values obtained from the peak temperature of tan δ are 325, 282 and 320 °C for Px–MMA/HP7200, x = 1–3 respectively. The *T*_g_ values obtained from the peak temperature of loss modulus (*E*″) are 297, 257, and 300 °C for Px–MMA/HP7200, x = 1–3 respectively. The values are relatively high, and are comparable with other epoxy thermosets. For property comparison, DMA thermogram of P1–OH/MMA was included in Figure 8. The *T*_g_ value of P1–MMA/HP7200 is even slightly higher than that of P1–OH/HP7200. Generally, active ester-cured epoxy thermosets exhibit lower *T*_g_ than that of phenolic-cured epoxy thermosets due to a reduction of intermolecular interaction. However, in this work, through the curing of the methacrylate double bond that increases the rigidity, no reduction in *T*_g_ was observed in P1–MMA/HP7200. Among the thermosets, P1–MMA/HP7200 and P3–MMA/HP7200 exhibit much higher *T*_g_ value than P2–MMA/HP7200. P1–MMA exhibits a bulky biphenylene phosphinate pendant and polar P=O bond that will hinder the rotation of polymer chains, so P1–MMA/HP7200 displays the highest *T*_g_ value. P3–MMA exhibits a polar trifluoromethyl moiety in the diamine structure unit, so P3–MMA/HP7200 displays a higher *T*_g_ than P2–MMA/HP7200, which has a less polar trimethyl moiety in the diamine structure unit. 

**Figure 9:**
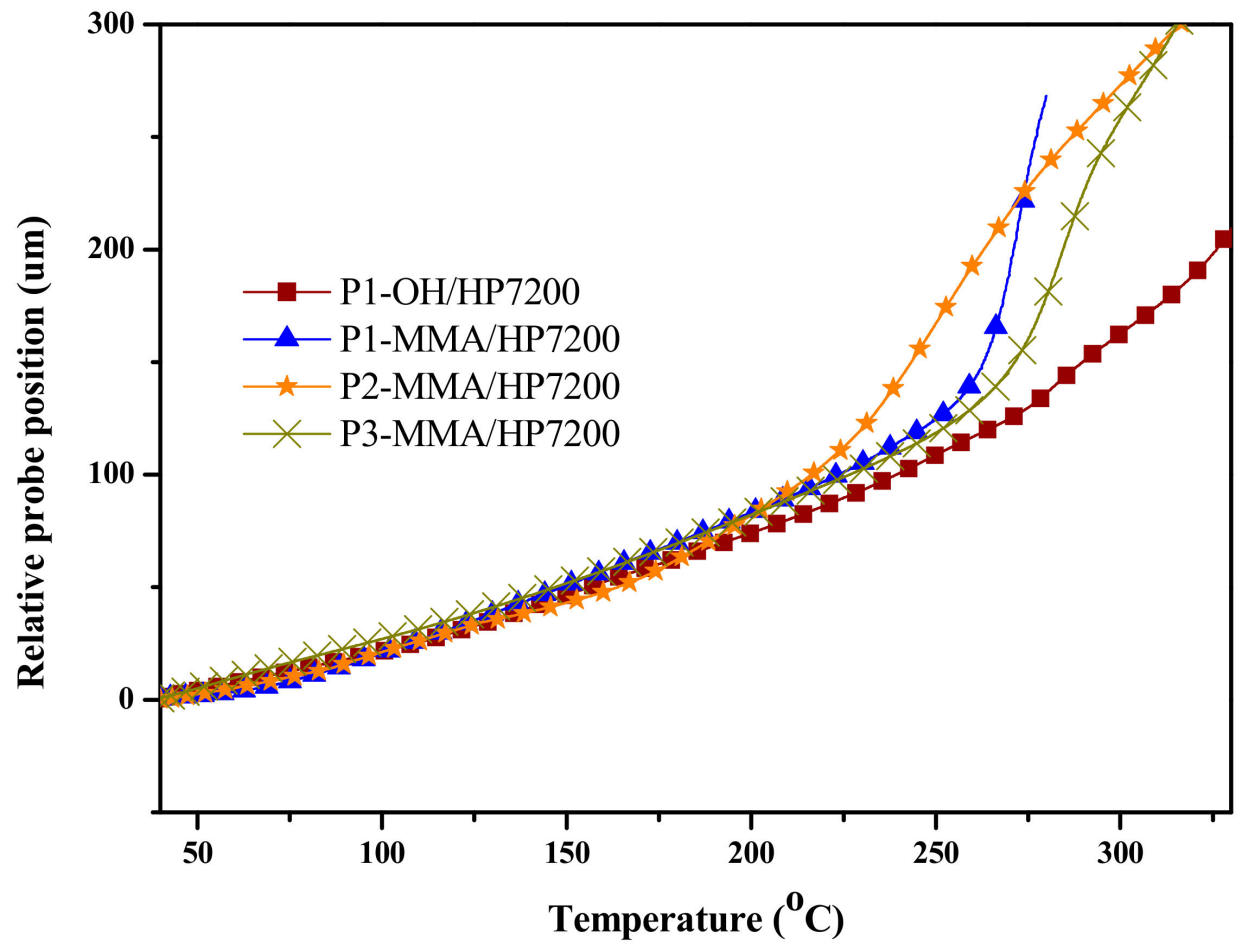
TMA thermograms of prepared polymers.

The high-*T*_g_ characteristic can also be confirmed by TMA measurement, as shown in Figure 9 and Table 2. The *T*_g_ values obtained from the onset temperature are 263, 227 and 274 °C for Px–MMA/HP7200, x = 1–3 respectively. The CTE value of P1–MMA/HP7200 is 48 ppm/°C, which is 7 ppm/°C smaller than that of P1–OH/HP7200. We think that the curing of methacrylate double bond that increases the rigidity explains the smaller CTE of P1–MMA/HP7200.

### 3.6. TGA Thermograms

The thermal stability was measured by TGA, and the results are listed in Table 2. The 5% decomposition temperature is above 430 °C in nitrogen atmosphere, and above 420 °C in air atmosphere. The char yield at 800 °C for P1–MMA/HP7200 is 47 and 16 wt.. % in nitrogen and air atmosphere, respectively, which are higher than that of P2–MMA/HP7200 and P3–MMA/HP7200. This result is thought to be related to the phosphorus element that formed thermally stable char at high temperature.

### 3.7. Contact Angle

The contact angle of prepared polymers was measured, and the results are listed in Table 3. The contact angles are 87.1°, 91.1°, and 92.2° for Px–MMA/HP7200, x = 1–3 respectively. The contact angle of P1–OH/ HP7200 is 68.7°, which is 18° smaller than that of P1–MMA/HP7200. The results suggest that P1–MMA/HP7200 is more hydrophobic than P1–OH/HP7200. The fact that the ester linkage in P1–MMA/HP7200 is more hydrophobic than the secondary alcohol in P1–OH/HP7200 explains the higher contact angle. In addition, the hydrogen boding with water may explain the lower hydrophobicity of P1–OH/HP7200. The trifluoromethyl further contribute the hydrophobic, so P3–MMA/HP7200 has the highest contact angle. Table 3 also shows the dielectric properties of prepared thermosets. The dielectric constants are 3.2, 3.0, and 2.9 U for Px–MMA/HP7200, x = 1–3 respectively. The dielectric value of 2.9 U is impressive compared with other epoxy thermosets (around 3.5 U) [44]. The dielectric constant and dissipation factor of P1–MMA/HP7200 are 3.2 U and 12.0 × 10^−3^ U, respectively, which are lower than those (3.4 and 16.7 × 10^−3^ U) of P1–OH/HP7200. The reason for the better dielectric properties is thought to be the same as for the contact angle. P2–MMA/HP7200 and P3–MMA/HP7200 shows higher contact angles and better dielectric properties than P1–MMA/HP7200, probably due to the existence of a polar P=O bond in P1–MMA/HP7200. The highest content of trifluoromethyl linkages in P3–MMA/HP7200 explains the highest contact angle and lowest dielectric properties of P3–MMA/HP7200 (2.9 and 10.2 × 10^−3^ U for dielectric constant and dissipation factor, respectively).

## 4. Conclusions

Three methacrylate-containing polyimides (Px–MMA, x = 1–3) were successfully prepared, characterized, and acted as curing agents for HP7200. The thermosetting films of Px–MMA/HP7200 (x = 1–3, respectively) are homogeneous and flexible. A six-step curing mechanism was proposed in Scheme 2 based on DSC, IR analyses, and the conclusions of three model reactions in our previous work. DMA and TMA thermograms show that Px–MMA/HP7200 exhibit very high-*T*_g_ characteristic compared with common epoxy thermosets. P1–OH/HP7200 was prepared and compared with P1–MMA/HP7200. The *T*_g_ value of P1–MMA/HP7200 is even slightly higher than that of P1–OH/HP7200. We propose that the curing of methacrylate double bond compensates the reduction in intermolecular interaction resulted from secondary alcohols. Among the thermosets, P1–MMA/HP7200 and P3–MMA/HP7200 exhibit much higher *T*_g_ value than P2–MMA/HP7200. P1–MMA exhibits a bulky biphenylene phosphinate pendant and polar P=O bond that will hinder the rotation of polymer chains, so P1–MMA/HP7200 displays the highest *T*_g_ value. P3–MMA exhibits a polar trifluoromethyl moiety in the diamine structure unit, so P3–MMA/HP7200 displays a higher *T*_g_ than P2–MMA/HP7200, which has a less polar trimethyl moiety in the diamine structure unit. P1–MMA/HP7200 exhibits a higher contact angle, and a lower dielectric constant and dissipation factor than P1–OH/HP7200. We propose that the ester linkages in P1–MMA/HP7200 is less polar than the hydroxyl groups in P1–OH/HP7200, explaining the more hydrophobic characteristic and better dielectric properties for P1–MMA/HP7200. The highest content of hydrophobic trifluoromethyl linkages in P3–MMA/HP7200 explains the highest contact angle and lowest dielectric properties of P3–MMA/HP7200 (2.9 and 10.2 × 10^−3^ U for dielectric constant and dissipation factor, respectively). The dielectric constant is 20% lower than that (about 3.5 U) of common epoxy thermosets. These epoxy thermosets might be applicable in high-frequency printed circuit boards. 

## Figures and Tables

**Table 1:** Effect of reaction conditions on the status of reaction for P1–MMA synthesis.

Run	Catalyst	Temperature (°C)	Time (h)	Status ^a^
1	DMAP (2 mole %)	100	12	Residual phenolic
2	DMAP (5 mole %)	100	12	Residual phenolic
3	Sodium acetate (1 wt. %)	100	12	Residual phenolic
4	Sodium acetate (1 wt. %)	80	12	Side reactions ^b^
5	DMAP (2 mole %)	80	12	Residual phenolic
6	DMAP (2 mole %)	50	12	Side reactions ^b^
7	DMAP (2 mole %)	50	24	Side reactions ^b^
8	DMAP (2 mole %)	25	24	Completed

^a^ Based on ^1^H-NMR spectrum of reaction product of P1–MMA, ^b^ No residual phenolic, but with side reactions (unknown peaks in the ^1^H-NNR spectrum).

**Table 2:** Thermal properties of prepared polymers.

Sample	*T*_g_ (°C) ^a^	*T*_g_ (°C) ^b^	*T*_g_ (°C) ^c^	CTE (10^−6^/°C) ^d^	*T*_d5%_ (°C) ^e^		Char Yield (%) ^f^	
					N_2_	Air	N_2_	Air
P1–OH/HP7200	323	288	266	55	464	466	43	19
P1–MMA/HP7200	325	297	263	48	444	460	47	16
P2–MMA/HP7200	282	257	227	50	431	421	26	0
P3–MMA/HP7200	320	300	274	54	433	433	41	0

^a^ Measured by DMA. *T*_g_ values were determined from the peak temperature of the tan δ curve. ^b^ Measured by DMA. *T*_g_ values were determined from the peak temperature of the loss modulus *E*″ curve. ^c^ Measured by TMA. *T*_g_ values were determined from the onset temperature of the TMA thermogram. ^d^ Coefficient of thermal expansion, recorded from 50 to 150 °C. ^e^ Temperature corresponding to 5% weight loss by thermogravimetry at a heating rate of 20 °C/min in nitrogen and air. ^f^ Residual weight % at 800 °C in nitrogen and air.

**Table 3:** Contact angle and dielectric properties of prepared polymers.

Sample	Contact Angle (Deg.)	Dielectric Constant	Dissipation Factor (×10^−3^)
P1–OH/HP7200	68.7	3.4 ± 0.005	16.7 ± 0.03
P1–MMA/HP7200	87.1	3.2 ± 0.003	12.0 ± 0.02
P2–MMA/HP7200	91.1	3.0 ± 0.004	11.0 ± 0.02
P3–MMA/HP7200	92.2	2.9 ± 0.005	10.2 ± 0.03
